# Phenome of coeliac disease vs. inflammatory bowel disease

**DOI:** 10.1038/s41598-022-18593-y

**Published:** 2022-08-26

**Authors:** Moritz Kleinjans, Carolin V. Schneider, Tony Bruns, Pavel Strnad

**Affiliations:** 1 Medical Clinic III, Gastroenterology, Metabolic Diseases and Intensive Care, University Hospital RWTH Aachen, Pauwelsstr. 30, 52074 Aachen, Germany; 2 The Institute for Translational Medicine and Therapeutics, The Perelman School of Medicine, University of Pennsylvania, Philadelphia, PA 19104 USA

**Keywords:** Inflammatory bowel disease, Coeliac disease

## Abstract

Coeliac disease (CeD) is characterized by gliadin-induced intestinal inflammation appearing in genetically susceptible individuals, such as HLA-DQ2.5 carriers. CeD, as well as other chronic intestinal disorders, such as Crohn's disease (CD) and ulcerative colitis, has been associated with increased morbidity and mortality, but the causes are unknown. We systematically analysed CeD-associated diagnoses and compared them to conditions enriched in subjects with CD/UC as well as in HLA-DQ2.5 carriers without CeD. We compared the overall and cause-specific mortality and morbidity of 3,001 patients with CeD, 2,020 with CD, 4,399 with UC and 492,200 controls in the community-based UK Biobank. Disease-specific phenotypes were assessed with the multivariable Phenome Wide Association Study (PheWAS) method. Associations were adjusted for age, sex and body mass index. All disease groups displayed higher overall mortality than controls (CD: aHR = 1.91[1.70–2.17]; UC: aHR = 1.32 [1.20–1.46]; CeD: aHR = 1.38 [1.22–1.55]). Cardiovascular and cancer-related deaths were responsible for the majority of fatalities. PheWAS analysis revealed 166 Phecodes overrepresented in all three disorders, whereas only ~ 20% of enriched Phecodes were disease specific. Seven of the 58 identified CeD-specific Phecodes were enriched in individuals homozygous for HLA-DQ2.5 without diagnosed CeD. Four out of these seven Phecodes and eight out of 19 HLA-DQ2.5 specific Phecodes were more common in homozygous HLA-DQ2.5 subjects with vs. without CeD, highlighting the interplay between genetics and diagnosis-related factors. Our study illustrates that the morbidity and mortality in CeD share similarities with CD/UC, while the CeD-restricted conditions might be driven by both inherited and acquired factors.

## Introduction

Chronic inflammatory intestinal diseases constitute a great burden for patients and health systems^1,2^. The most relevant entities include Crohn's disease (CD), ulcerative colitis (UC) and coeliac disease (CeD). CD and UC share many similarities and are therefore often summarized as inflammatory bowel disease (IBD). They have a multifactorial aetiology characterized by a dysfunctional intestinal barrier and dysregulated immune responses^3–5^. While UC is limited to the colon, CD can affect the entire gastrointestinal tract and typically includes the terminal ileum^4,5^. Patients with CD often develop complications such as abscesses and fistulas, while colorectal carcinoma constitutes a characteristic complication of UC^4^.

CeD is an immune-mediated enteropathy causing chronic duodenal inflammation in genetically predisposed individuals. It is triggered by exposure to gliadin, a widely spread dietary component^2^. In addition to intestinal symptoms such as abdominal pain, diarrhoea, and malabsorption, CeD is also associated with extraintestinal comorbidities, including cardiovascular and haematologic diseases, as well as many diseases from the autoimmune spectrum^6–8^. The main genetic predisposition for the development of CeD is the presence of susceptible human leukocyte antigens (HLAs), i.e., cell-surface proteins responsible for the regulation of the immune system. Among them, HLA-DQ2 or -DQ8 are able to bind gluten-derived peptides and present them to CD4 + T cells^9^. Most patients with CeD carry one or more of the disease-related HLA alleles DQ2.5, DQ2.2, or DQ8, but the presence of these variants is not sufficient to develop CeD^2,7^. The DQ2.5 variant is the most important genetic factor, occurring in approximately 90% of CeD patients^9^. Since an apparent gene-dose effect exists, patients carrying the homozygous HLA-DQ2.5 allele are at particularly high risk for CeD^7,9^. However, other factors, including non-HLA genetic risk factors and changes in the microbiome, might play a role in CeD, and the same is true for CD/UC^10–12^.

Although CeD is deemed less life-threatening than CD and UC, several studies suggest that it is associated with excess mortality^13–16^. The underlying mechanisms remain unclear, but they might be related to the presence of acquired (i.e., diagnosis-related) factors, genetic background, or both. To address this issue, we used the UK Biobank and compared the overall and cause-specific mortality and morbidity of patients with CeD and IBD. The goal of our study was to identify how many CeD-associated disorders are CeD-specific and how many are shared with CD/UC. Finally, we wondered whether the CeD-specific Phecodes are related to the genetic background of CeD subjects or are instead associated with diagnosis-related factors.

## Results

### Overall- and cause-specific mortality of analysed intestinal diseases

Among the 502,488 individuals recruited to UKB, 3001 were diagnosed with coeliac CeD, 4,399 with UC, and 2020 with CD, while 868 subjects with more than one diagnosis of CeD, UC and CeD were excluded.

A total of 492,200 individuals had none of these diagnoses and were used as a reference group (Supplementary Fig. S1A). All subgroups had a similar age distribution at baseline, while CeD subjects had the highest proportion of women and the lowest average body mass index (Table 1).

**Table 1:** Baseline characteristics of the study population.

	Reference (n = 492,200)	Coeliac disease [K90] (n = 3001)	Ulcerative Colitis [K51] (n = 4399)	Crohn’s disease [K50] (n = 2020)
Age (years)	56.5 ± 8.1	57.8 ± 7.8	57.6 ± 7.9	56.82 ± 8.1
Males	224,490 (45.6%)	1046 (34.9%)	2293 (52.1%)	872 (43.2%)
BMI (kg/m^2^)	27.4 ± 4.8	26.2 ± 4.8	27.6 ± 4.7	27.2 ± 5.0

The reference group consists of individuals without Crohn´s disease, ulcerative colitis, or coeliac disease.

*BMI* body mass index.

During the median follow-up of 12 years, 271 CeD patients (9.0%), 437 UC patients (9.9%), 250 CD patients (12.4%), and 32,934 participants from the reference group (6.7%) died (Supplementary Fig. S2). Patients harbouring either one of the intestinal disorders displayed increased overall mortality compared to the reference group (Supplementary Fig. S2), and the elevated death rate remained significant after adjustment for age, sex and BMI. Subjects with CD had the highest overall mortality rate (aHR = 1.91[1.70–2.17]), while the overall mortality rates of CeD and UC individuals were comparable (CeD: aHR = 1.38 [1.22–1.55]; UC: aHR = 1.32 [1.20–1.46]) (Table 2).

**Table 2:** Overall and disease-specific mortality in subjects with analysed intestinal disorders.

Cause of death	Coeliac disease [K90] (n = 3001)				Ulcerative colitis [K51] (n = 4399)				Crohn’s disease [K50] (n = 2020)			
	n	deaths/1000 PYs	HR	95%-CI	n	deaths/1000 PYs	HR	95%-CI	n	deaths/1000 PYs	HR	95%-CI
Overall mortality	271	7.72	**1.38**	1.22–1.55	437	8.49	**1.32**	1.20–1.46	250	10.67	**1.91**	1.70–2.17
Malignancies (C00-C97)	129	3.67	**1.22**	1.03–1.45	205	3.98	**1.25**	1.09–1.43	108	4.61	**1.60**	1.33–1.93
Digestive diseases (K00-K93)	17	0.48	**2.58**	1.60–4.16	37	0.72	**3.14**	2.26–4.35	30	1.28	**6.45**	4.49–9.26
Cardiovascular diseases (I00-I99)	51	1.45	**1.41**	1.07–1.86	88	1.71	1.21	0.98–1.50	50	2.13	**1.90**	1.44–2.52
Respiratory diseases (J00-J99)	25	0.71	**1.76**	1.18–2.61	32	0.62	1.32	0.93–1.88	21	0.90	**2.29**	1.49–3.52

Multivariable hazard ratios were determined for patients with coeliac disease, ulcerative colitis, and Crohn’s disease compared to the reference group that displays none of these conditions.

All analyses were adjusted for age, sex and body mass index.

For details, see Supplementary Table S4.

*PYs* person-years, *CI* confidence interval, *HR* hazard ratio.

Significant values are in bold.

In all three intestinal disorders, cancer and cardiovascular diseases were responsible for the majority of fatalities (63–67%), whereas digestive diseases accounted for only 6–12% of deaths, with the lowest rate in CeD. Cancer- and digestive disease-specific mortality was elevated in all disorders, while cardiovascular and respiratory mortality was increased in CeD and CD but not UC subjects (Table 2). Overall, the pattern of disease-specific mortalities was similar in all three cohorts.

### PheWAS analysis reveals disease-specific phenotypes

**Figure 1:**
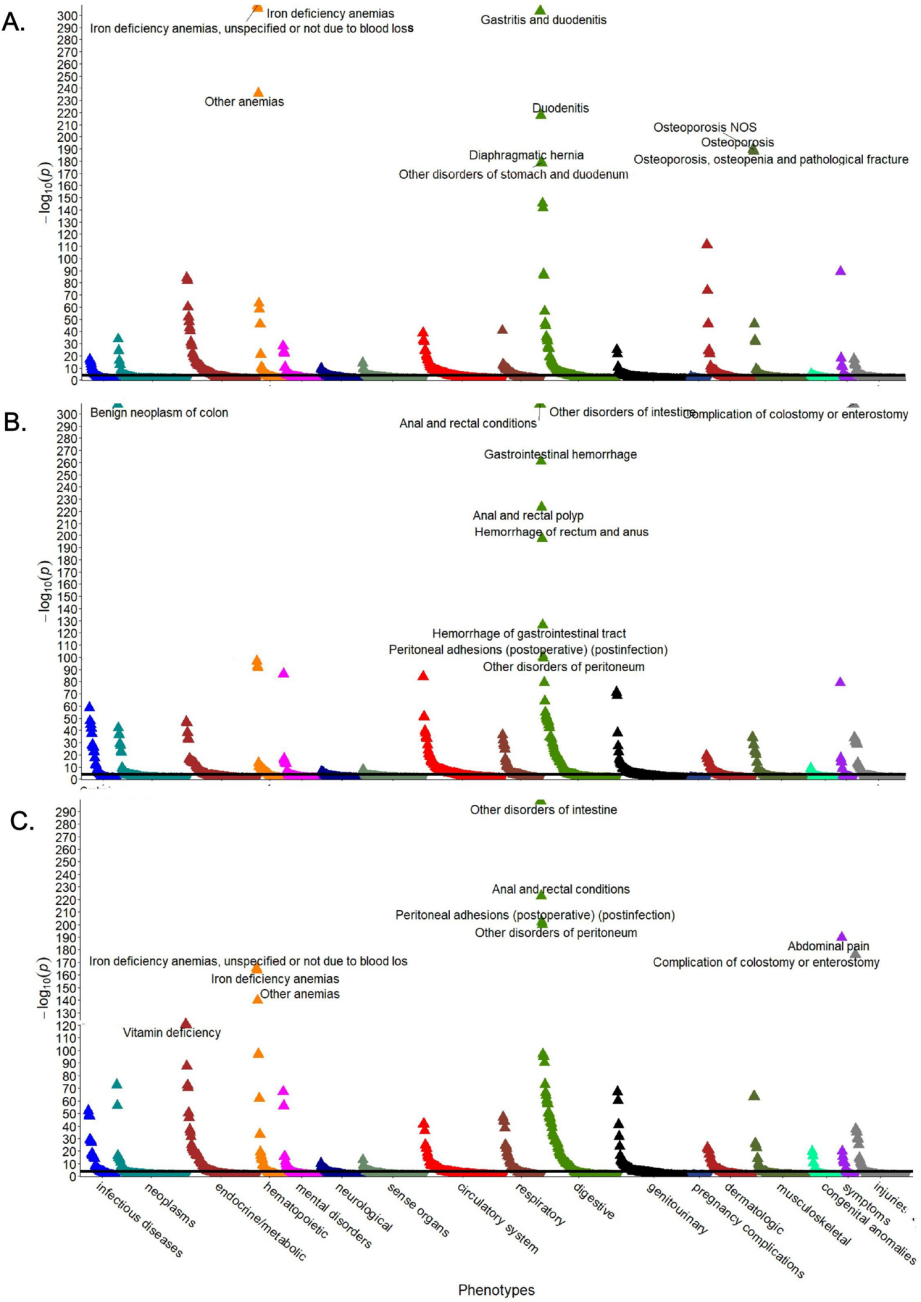
Conditions associated with CeD and IBD. Manhattan plots display Phecodes that are significantly over/underrepresented in individuals with coeliac disease (**A**), ulcerative colitis (**B**), or Crohn´s disease (**C**) compared with the reference group without these diagnoses. The ten most significant associations are shown. Upwards/downwards pointing triangles refer to Phecodes that are over/underrepresented. The black line indicates the significance level after Bonferroni adjustment for multiple testing. All analyses were adjusted for sex, age, and body mass index, and p values are displayed in a − log10 format. *NOS* not otherwise specified.

**Figure 2:**
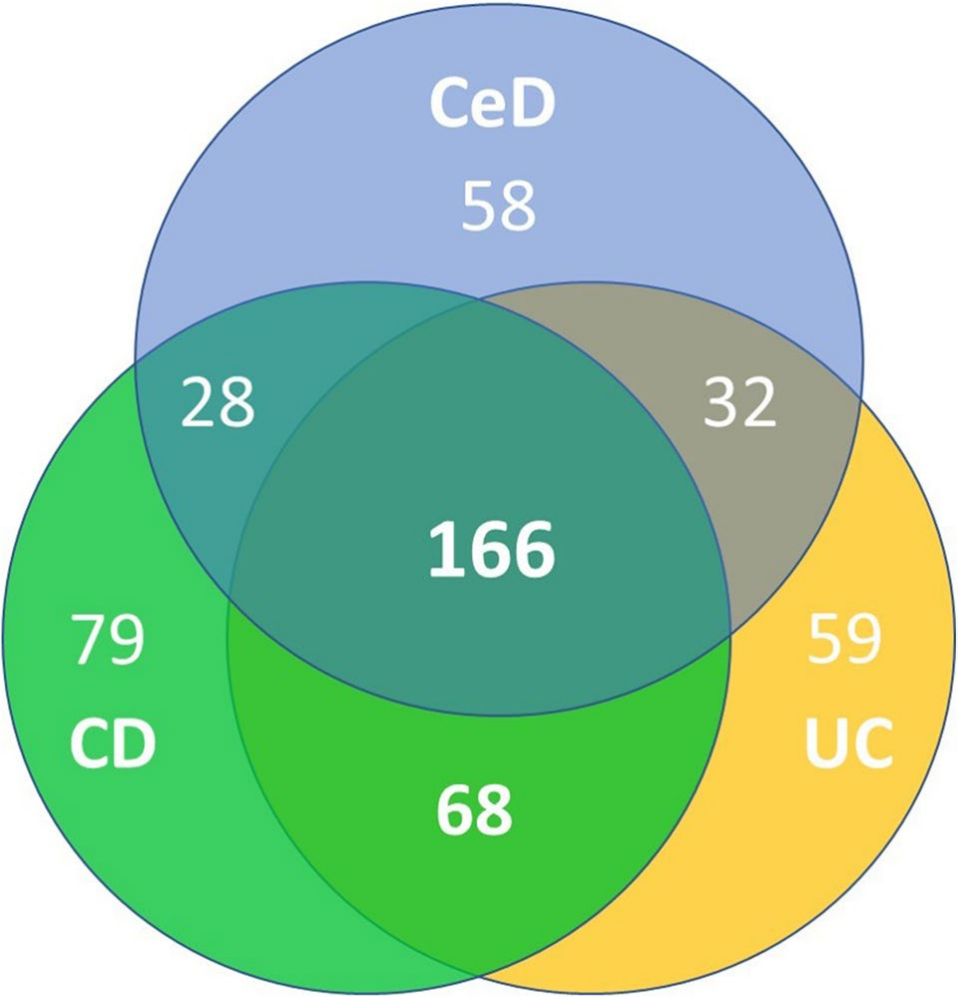
Overview of Phecodes associated with the analysed intestinal disorders. The Venn diagram depicts the numbers of conditions associated with coeliac disease (CeD), Crohn´s disease (CD), and ulcerative colitis (UC). The results are based on the PheWAS analysis comparing the occurrence of all available conditions in individuals with the displayed intestinal disorder versus the reference group without any of these intestinal diseases adjusted for multiple testing.

To decipher whether the observed comorbidities represent a conserved response to chronic intestinal injury or whether they constitute unique, disease-specific traits, we performed multivariable PheWAS analysis comparing the different intestinal diseases with the reference group (Fig. 1). Among all investigated intestinal disorders, an enrichment of Phecodes for gastrointestinal, haematopoietic and metabolic diseases, symptoms, and complications was observed. Osteoporosis, anaemia, and dermatological Phecodes were more prominent in patients with CeD than in patients with IBD (Fig. 1). The phenotypic overlap between the intestinal disorders became apparent in the Venn diagram, which revealed 166 Phecodes shared between all three disorders (Fig. 2). The number of shared Phecodes between CD and UC was significantly greater than that between CeD and one of the two inflammatory bowel disease subgroups (CeD and CD: 28; CeD and UC: 32; CD and UC: 68). The number of Phecodes specific to each disease was comparable for CeD and UC (CeD: 58, UC: 59), while CD had the highest number of unique Phecodes (CD: 79) (Fig. 2). Notably, only 18–23% of associated Phecodes were disease specific, while the others were shared by at least two disorders and 49–58% by all three disorders (Fig. 2).

### Autoimmune phecodes are enriched in CeD

**Figure 3:**
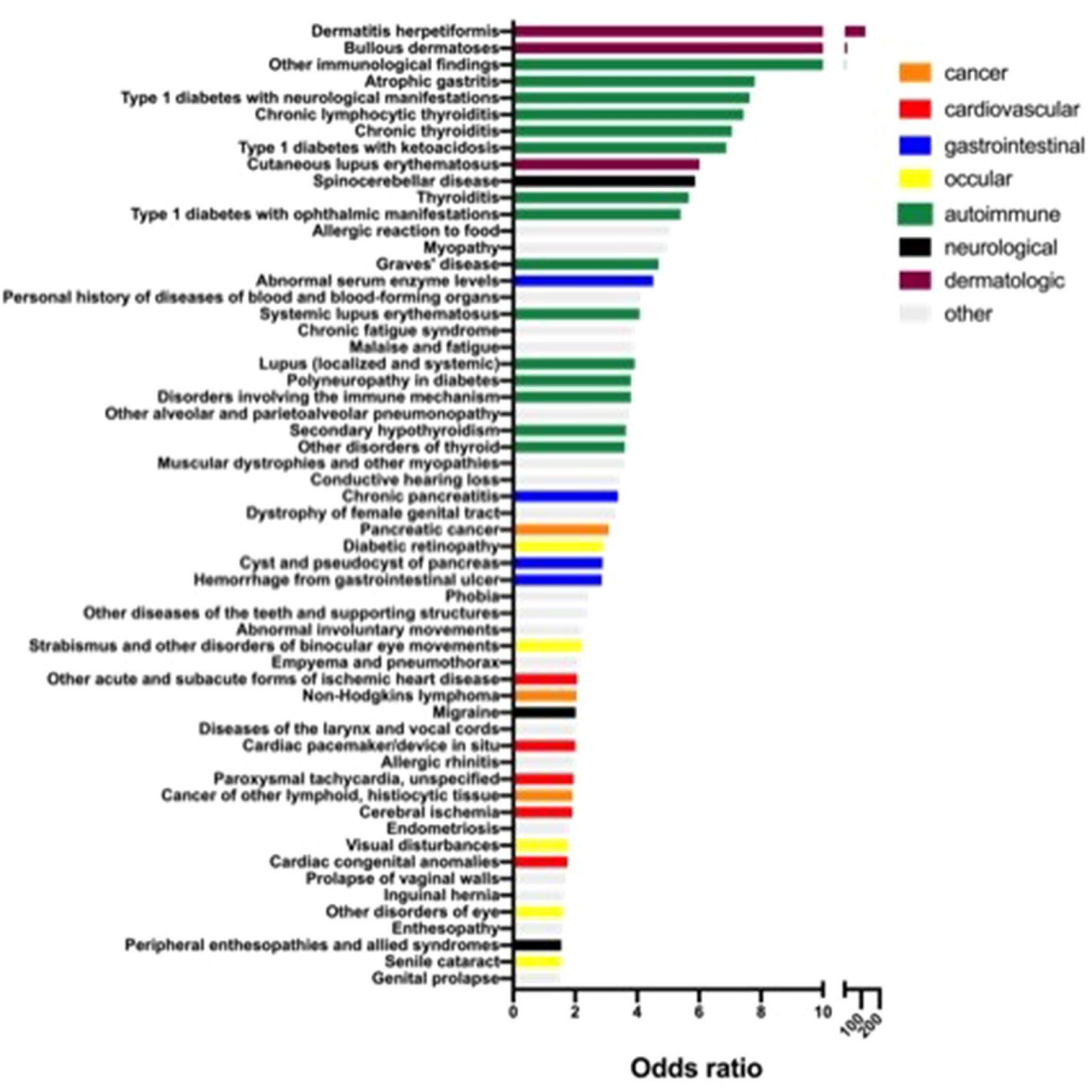
Overview of Phecodes significantly overrepresented in individuals with coeliac disease. Only Phecodes enriched in patients with coeliac disease but not in Crohn's disease or ulcerative colitis are shown. Odds ratios indicate the occurrence of corresponding Phecodes in subjects with coeliac disease compared with the reference group that carries none of the analysed intestinal disorders. Colours highlight the indicated disease classifications of Phecodes as described in the figure.

To delineate conditions associated with CeD, we assessed CeD-specific Phecodes (Fig. 3). Among them, Phecodes of well-established CeD-associated dermatologic manifestations such as "dermatitis herpetiformis" (OR = 122.2) and "bullous dermatoses" (OR = 23.6) displayed the highest odds ratios. Notably, Phecodes of autoimmune diseases were particularly prominent. They included diabetes and thyroid disorders (Fig. 3) and displayed odds ratios ranging from 3.6 ("other disorders of thyroid") to 14.3 ("other immunological findings"). Several cardiovascular and ocular disorders were also enriched in patients with CeD, with odds ratios of approximately 2. The only Phecodes coding for malignancy that were significantly enriched in patients with CeD were "pancreatic cancer" (OR = 3.1), "non-Hodgkin´s lymphoma” (OR = 2.0), and “malignancies of other lymphatic tissue” (OR = 1.9). As potential reasons for the relatively high respiratory disease-specific mortality, CeD individuals were more likely to harbour “other alveolar and parietoalveolar pneumonopathy” as well as “empyema and pneumothorax”. Finally, gastrointestinal diseases played only a minor role among the CeD-specific Phecodes. In contrast, digestive and genitourinary conditions were more commonly seen among CD-/UC-specific Phecodes, while infectious, autoimmune and ocular codes were relatively rare (Supplementary Figs. S3, S4). Moreover, digestive disorders were highly enriched among conditions that are overrepresented both in UC and CD but not CeD individuals (18 out of 68 Phecodes). Notably, the latter subgroup also contained 9 infection-related Phecodes (Supplementary Table S3).

**Figure 4:**
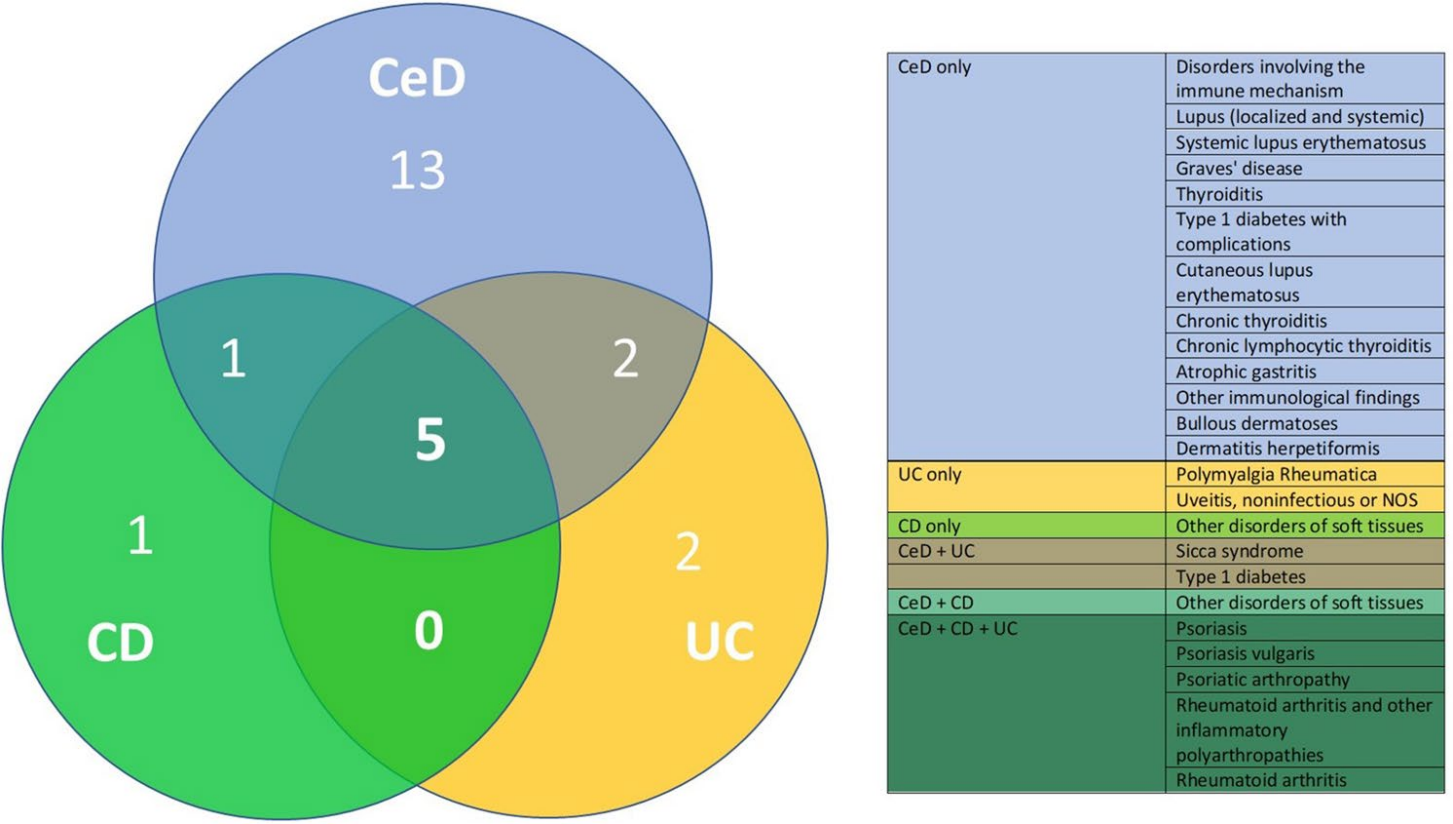
Overview of autoimmune Phecodes associated with the analysed intestinal disorders. Venn diagram depicts the numbers of autoimmune conditions associated with coeliac disease (CeD), Crohn´s disease (CD), and ulcerative colitis (UC). The results are based on the PheWAS analysis comparing the occurrence of all available Phecodes in individuals with the displayed intestinal disorder versus the reference group without any of these intestinal diseases. All conditions are listed in a colour-coded manner in the accompanying table. *NOS* not otherwise specified.

Since Phecodes of autoimmune diseases constituted the most frequent disease spectrum enriched in subjects with CeD, we analysed whether autoimmune Phecodes were significantly associated with UC and CD as well. The corresponding Venn diagram revealed that five Phecodes were overrepresented in all intestinal disorders, with all of them being related to rheumatoid arthritis and psoriasis. One and two unique autoimmune Phecode(s) were more enriched in CD and UC individuals, respectively, compared to 13 in CeD subjects (Fig. 4).

### HLA-DQ2.5-dependent Phecodes and the impact of CeD diagnosis on the appearance of HLA-specific Phecodes

**Figure 5:**
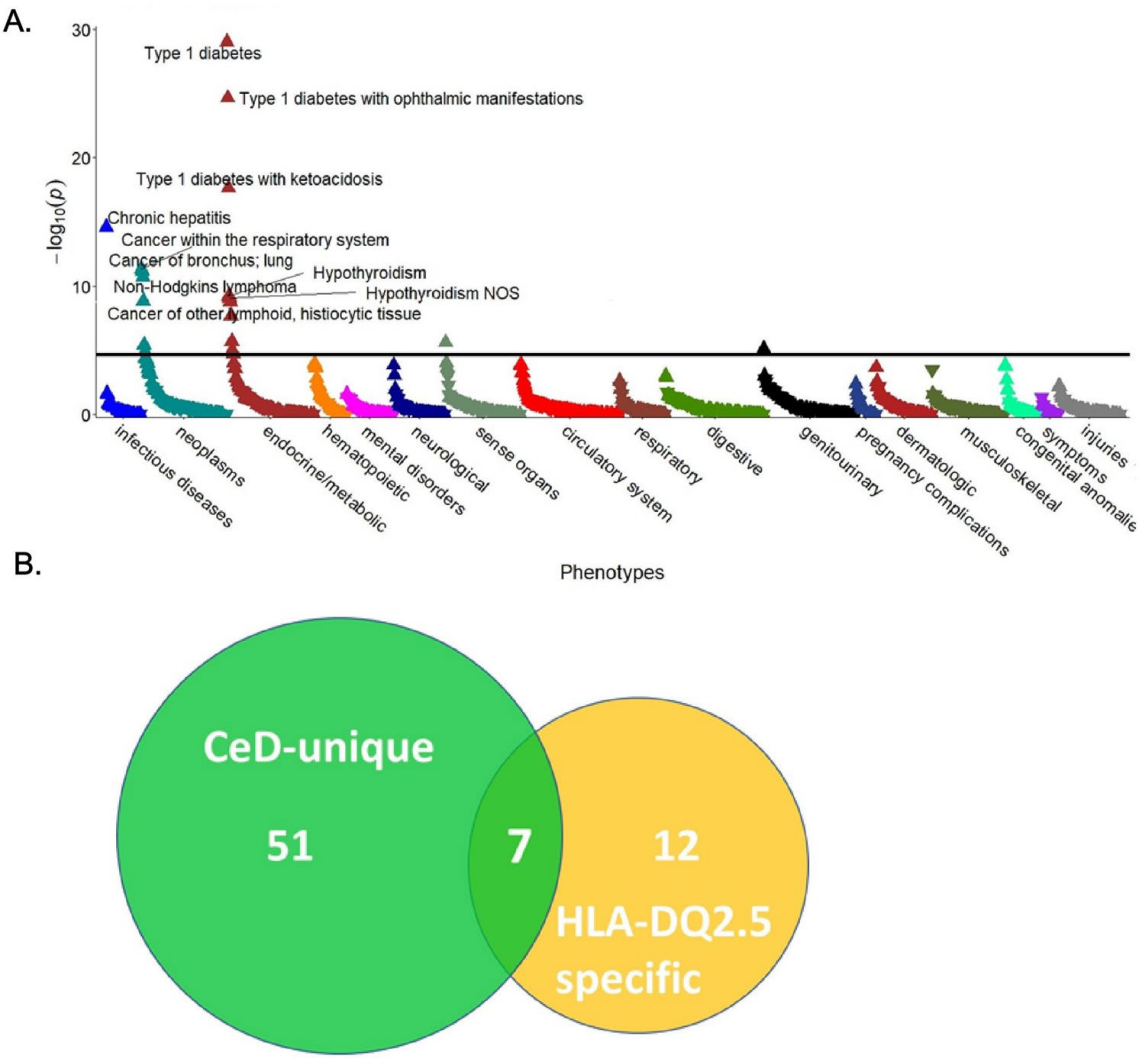
Conditions associated with two versus 0–1 HLA-DQ2.5 alleles in individuals without coeliac disease and their overlap with CeD-specific Phecodes. (**A**) The Manhattan plot highlights Phecodes that are significantly over/underrepresented as upwards/downwards pointing triangles. The ten most significant associations are shown. The black line indicates the significance level after Bonferroni adjustment for multiple testing. All analyses were adjusted for sex, age and body mass index, and p values are displayed in a − log10 format. (**B**) A Venn diagram illustrating the number of Phecodes specifically enriched in coeliac disease (CeD) subjects vs. controls (green) and overrepresented in noncoeliac patients with two vs. 0–1 HLA-DQ2.5 alleles (yellow). *NOS* not otherwise specified.

**Figure 6:**
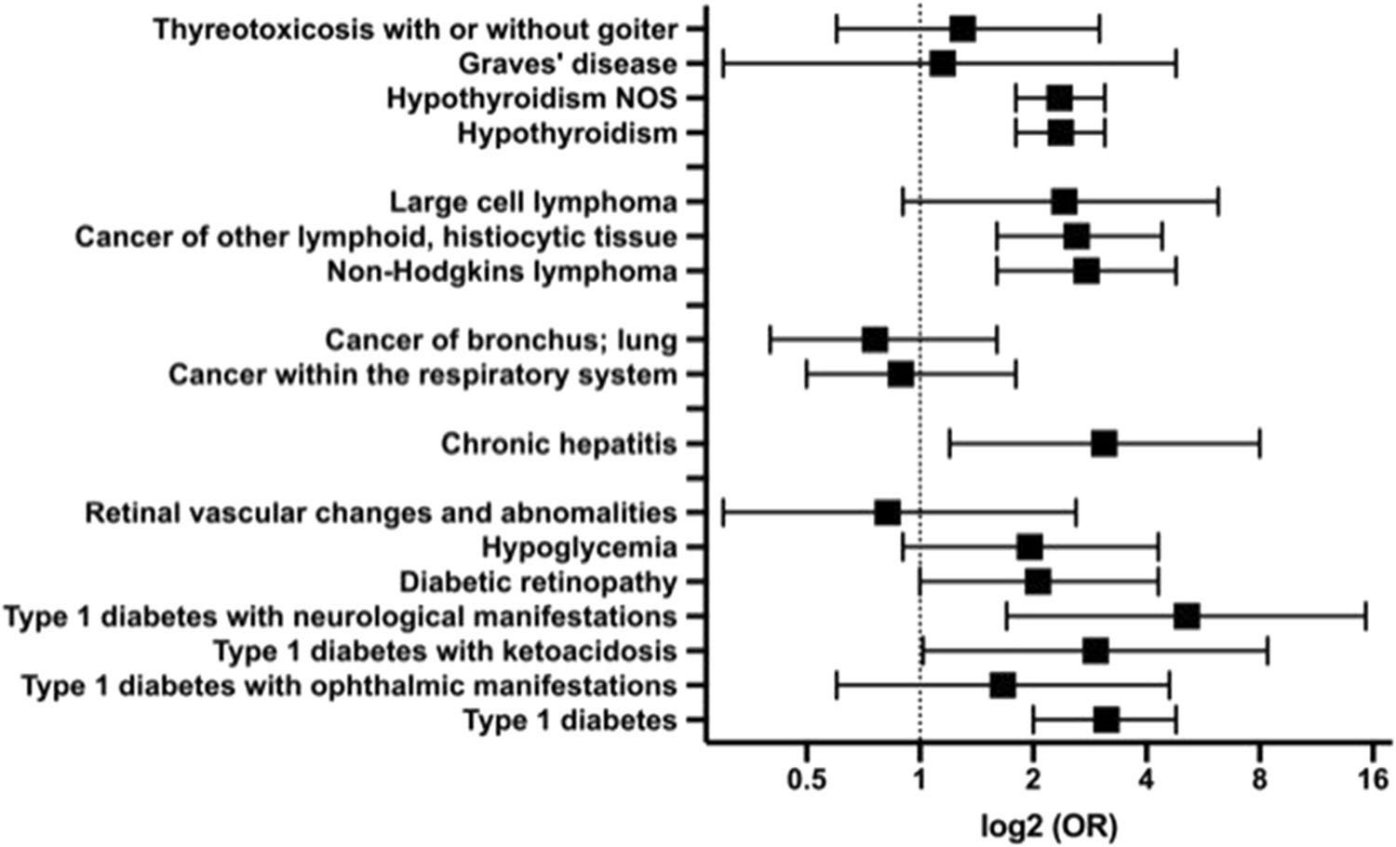
Impact of the presence of coeliac disease on Phecodes associated with HLA-DQ2.5 homozygosity. The occurrence of the highlighted Phecodes was compared in HLA-DQ2.5 homozygous individuals with vs. without the diagnosis of coeliac disease. Odds ratios (ORs) and the corresponding 95% confidence intervals are shown. Nonproliferative glomerulonephritis and tongue cancer were not included since they were not present in the group of CeD patients with HLA-DQ2.5 homozygosity. *NOS* not otherwise specified.

As CeD is highly associated with specific HLA genotypes, we studied whether the underlying genetic background may contribute to the observed CeD-specific Phecodes. To this end, we focused on subjects with two DQ2.5 alleles, which are strongly predisposed to CeD (Supplementary Fig. S1B). A multivariable analysis restricted to patients without the diagnosis of CeD (Fig. 5A, Supplementary Fig. S1B) revealed that 19 Phecodes were overrepresented in individuals with two vs. 0–1 HLA-DQ2.5 alleles (Supplementary Table S1). The largest changes were observed in the categories “neoplasms” and “endocrine metabolic” (Fig. 5A). Significantly enriched Phecodes of malignant diseases comprised tumours of the respiratory system (OR = 1.6), “cancer of tongue” (OR = 2.6), “non-Hodgkin’s lymphoma” (OR = 1.9), “large cell lymphoma” (OR = 2.0), and “cancer of other lymphatic, histiocytic tissue” (OR = 1.7). Among Phecodes encoding endocrine diseases, diabetes mellitus type 1 and its complications as well as thyroid disorders, including “hypothyroidism”, “Graves´ disease,” and “thyrotoxicosis”, were the most prominent (Supplementary Table S1). In addition, “chronic hepatitis” (OR = 3.8) and “nonproliferative glomerulonephritis” (OR = 3.0) were also significantly enriched in subjects with a genetic predisposition to, but without the concomitant diagnosis of, CeD (Fig. 5A, Supplementary Table S1). Out of the 19 Phecodes enriched in noncoeliac DQ2.5 homozygotes, seven were also uniquely enriched in CeD, i.e., were overrepresented in CeD subjects but not UC/CD individuals (Fig. 5B). These included diabetes and its complications, “Graves´ disease”, “non-Hodgkin lymphoma,” and “cancer of lymphoid, histiocytic tissue”. This finding prompted us to investigate whether the observed HLA-associated Phecodes were further affected by the presence of CeD. To that end, we compared HLA-DQ2.5 homozygous individuals with and without the diagnosis of CeD (Fig. 6, Supplementary Table S2). Eight Phecodes were significantly enriched in the former group, while none of them was less frequent. These included “type 1 diabetes” (OR = 3.1) together with its complications, “hypothyroidism” (OR = 2.4), “chronic hepatitis” (OR = 3.1), and “non-Hodgkin’s lymphoma” (OR = 2.8). In contrast, the frequency of respiratory tract cancer and thyrotoxicosis/Graves´ disease was similar in HLA-DQ2.5 homozygous subjects with and without the diagnosis of CeD (Fig. 6, Supplementary Table S2). In conclusion, while some of the CeD-specific Phecodes were primarily driven by the HLA dosage, the majority might be HLA-independent or driven by a combination of genetic susceptibility and the presence of diagnosis-related factors.

## Discussion

Our study demonstrated that all three analysed intestinal disorders are associated with overall excess mortality, with CD conferring the highest hazard ratio. Although this was suggested previously^13,17,18^, the risks seen in the UKB cohort somewhat exceed the numbers reported in large meta-analyses. In all three diseases, cancer- and cardiovascular-related deaths accounted for the majority of the cases, and the specific death risks closely resembled the overall mortality for the corresponding intestinal disorder. While increased cancer-related mortality has been established for CD^18,19^, the data on other disorders and the association between intestinal inflammation and cardiovascular mortality are conflicting, and further studies are needed to clarify the discrepant observations^13,19–22^. The excess digestive disease-related deaths that were particularly overrepresented in CD are well in line with published findings^13,22–24^. They, however, accounted for only a minority of cases and did not explain the elevated overall death rate. In summary, our data suggest that the presence of chronic intestinal disease, irrespective of its aetiology, increases overall mortality and that the predisposition to the most common causes of death, i.e., cardiovascular and malignant diseases, plays a significant role. This is reminiscent of rheumatoid arthritis, another chronic inflammatory disorder that is associated with increased cardiovascular mortality^25^.

The similarities of intestinal inflammatory disorders seen on the mortality level were confirmed in PheWAS analysis showing that ~ 50% of all associated Phecodes were shared by all three conditions, while only ~ 20% were specific for one of these three diseases. Autoimmune disorders were particularly prominent in CeD, which is compatible with previous reports^26,27^. The marked association between CeD and autoimmune disorders is likely related to CeD's most established immune pathomechanisms and the strongest HLA association^28^. In this respect, we clearly demonstrated that some of the CeD-specific disorders, such as type 1 diabetes, thyroid disorders including Graves´ disease, and non-Hodgkin lymphoma, are affected not only by the CeD itself but also by the associated genetic background. This is well in line with the association of type 1 diabetes, Graves´ disease, and non-Hodgkin lymphoma with specific HLA haplotypes reported in the literature^29–31^. Taken together, our findings both confirm and extend previous findings.

The major aim of our study was to shed some light on the interplay between genetic background and acquired factors in the development of CeD-related disorders. To this end, we specifically analysed the occurrence of HLA-DQ2.5-related Phecodes in HLA-DQ2.5 homozygous individuals with or without CeD. We demonstrated that eight out of 19 identified Phecodes were further enriched in homozygous HLA-DQ2.5 individuals with versus without CeD. This suggests that the presence of CeD-related factors promotes the formation of autoreactive immune cells and further amplifies genetic risks. This concept was previously reported in the literature and has been (among others) proposed to play a crucial role in the development of non-Hodgkin lymphoma, type 1 diabetes, and coeliac hepatitis^32–34^.

Intestinal disease may also increase the susceptibility to other inflammatory diseases, such as rheumatoid arthritis and psoriasis, as these diseases are associated with all three intestinal disorders. Notably, IBD, psoriasis and rheumatoid arthritis display alterations in similar inflammatory pathways involving, among others, Th17 cells^35,36^, and are targeted by comparable anti-inflammatory treatment strategies^4,5,37,38^. Although immunosuppressive drugs are not used for coeliac disease, the involvement of Th17 cells has been demonstrated^36,39^, and multiple reports describe an association between CeD and rheumatoid arthritis as well as psoriasis^40–42^. However, the observed association of all analysed intestinal disorders with rheumatoid arthritis might be confounded by the difficult discrimination from enteropathic arthritis^43^.

A major limitation of the study is its cohort design, which precludes the identification of causal relationships. Moreover, in the UK Biobank cohort, the diagnosis of CeD is based on ICD10 codes, and no histological data are available. The prevalence of CeD in the UK biobank is only approximately half of what would be expected from prevalence studies (~ 1%)^44^. This suggests that some cases remain undiagnosed and/or unreported, which is in line with previous population-based studies^45^. This can lead to the overrepresentation of more severe CeD cases and might be partly responsible for the high overall mortalities seen in our study. However, several well-performed studies used the same approach to define participants with CeD and observed a similar performance as other cohort studies^46,47^. In contrast, the prevalence of CD/UC subjects meets/exceeds the rates reported in other studies, which suggests a satisfactory diagnostic rate. Another important limitation is that due to the complexity of CeD, we were not able to address all potential contributors, such as the role of further genetic variants or acquired factors, such as intestinal leakage, chronic immune cell activation, dietary composition or the microbiome. Because of the former, the importance of genetic factors might be larger than suggested by our analysis. Further studies are needed to delineate the exact origin of CeD-associated Phecodes and to determine which conditions directly contribute to the increased mortality seen in CeD.

## Methods

### Study cohort

The 'UK Biobank' is a population-based cohort study that enrolled approximately 500,000 participants aged 37–73 years at 22 recruiting centres all over the United Kingdom^48^. At the baseline visit (2006–2010), all participants gave consent for genotyping and data linkage to medical reports. Physical measurements and demographic information were obtained, and medical reports from 1996 onwards were used to record ICD-10 diagnoses. This source was used to identify patients with CeD (K90.0), UC (K51), and CD (K50) for our analysis.

The UK Biobank is linked to the national death register, which provided age at death and primary ICD-10 diagnosis that led to death for all participants who died during or prior to April 2021. The end of follow-up was defined as death or the end of hospital inpatient data collection in April 2021. Causes of death were grouped according to their ICD codes into malignancies (C00-C97), digestive diseases (K00-K93), cardiovascular diseases (I00-I99), and respiratory diseases (J00-J99).

For the first 50,000 subjects, genotyping was performed using the UK BiLEVE Axiom Array, while the Affymetrix UK Biobank Axiom Array was used for the remaining 450,000 participants. HLA-DQ2.5 status was determined using the SNP rs2187668.

The study was approved by the UKB Access Committee (Project #47527). All participants provided written consent for the study. The UK Biobank study has approval from the Northwest Multicentre Research-Ethics Committee. The manuscript is based solely on the analysis of pseudonymized data obtained from the UK Biobank Resource under Application Number 47527. The authors were not in contact with the described individuals, nor had they access to their personal data. We confirm that all research was performed in accordance with the Declaration of Helsinki and other relevant guidelines. The data were reported as described by the STROBE guidelines.

### PheWAS analysis

Participants were assigned to the disease-specific subgroups based on their ICD-10 diagnoses. Subjects with more than one of the analysed conditions (i.e., CeD, UC, or CD) were excluded (n = 868). The remaining participants were used as the reference cohort (Supplementary Fig. S1). ICD-10 diagnoses obtained from medical reports were collected for each subject, and duplicates were removed. To perform a PheWAS analysis, all ICD 10-codes were converted into Phecodes using the "PheWAS" R package^49,50^. Phecodes represent a high-throughput phenotyping tool used to rapidly define the case/control status of clinically meaningful diseases and conditions^48^. Using this package, a series of case–control tests were performed: (1) each analysed case group was generated by including patients with the corresponding Phecode; (2) individuals were assigned to the control group when they lacked the tested Phecode; and (3) to ensure statistical power, analysis was restricted to Phecodes with at least 200 cases^51^. Autoimmune Phecodes were identified using the official list of the American autoimmune association^52^.

### Statistical analysis

All continuous variables are presented as the mean ± standard deviation. Categorical variables are displayed as absolute and relative frequencies. Odds and hazard ratios are presented with their corresponding 95% confidence intervals (CIs). Hazard ratios were calculated using Cox proportional hazard regression models. Mortality was depicted as deaths per 1000 person-years, which were calculated using the following formula: (number of deaths/total number of subjects)/mean survival * 1000. To test for independent associations, multivariable logistic regression was used. All multivariable analyses were adjusted for age, sex and body mass index (BMI). PheWAS analysis was performed using the "PheWAS" R package. Bonferroni correction was used to adjust for multiple testing, and differences were considered to be statistically significant when p < 0.05. The data were analysed using SPSS Statistics version 27 (IBM; Armonk, NY, USA) and visualized with Prism version 8 (GraphPad, La Jolla, CA, USA).

## Supplementary Information

Supplementary Information.

## Data Availability

The data underlying this article are part of the UK Biobank database (https://www.ukbiobank.ac.uk/) and can be accessed after prior application. This research has been conducted under Application Number 47527.
