# Supplementary material for: Phenome of coeliac disease vs. inflammatory bowel disease
Source: Sci Rep. 2022 Aug 26;12:14572. doi: 10.1038/s41598-022-18593-y (PMC9418215; doi:10.1038/s41598-022-18593-y)
Supplement: Supplementary file 1 — Supplementary Information. [file 41598_2022_18593_MOESM1_ESM.docx]

**Supplementary Material of**

**Phenome of coeliac disease vs. inflammatory bowel disease**

Moritz Kleinjans^1,#^, Carolin V. Schneider^1,2,#^, MD, Tony Bruns^1^, MD, Pavel Strnad, MD^1,*^

**Supplementary Table 1. PheCodes significantly enriched in non-coeliacs with two vs. 0-1 HLA-DQ2.5 alleles.** PheCodes are shown with their corresponding odds ratios (OR) and 95% confidence intervals (95% CI). All analyses were adjusted for age, sex and body mass index.

| **PheCode - number** | **Phecode - name** | **OR** | **95%-CI** |
| --- | --- | --- | --- |
| 250.1 | Type 1 diabetes | 2.17 | 1.9-2.5 |
| 250.13 | Type 1 diabetes with ophthalmic manifestations | 3.74 | 2.9-4.9 |
| 250.11 | Type 1 diabetes with ketoacidosis | 4.39 | 3.1-6.2 |
| 70.4 | Chronic hepatitis | 3.79 | 2.7-5.4 |
| 165 | Cancer within the respiratory system | 1.61 | 1.4-1.8 |
| 165.1 | Cancer of bronchus; lung | 1.64 | 1.4-1.9 |
| 202.2 | Non-Hodgkins lymphoma | 1.85 | 1.6-2.2 |
| 244 | Hypothyroidism | 1.27 | 1.2-1.4 |
| 244.4 | Hypothyroidism NOS | 1.28 | 1.2-1.4 |
| 202 | Cancer of other lymphoid, histiocytic tissue | 1.66 | 1.4-1.9 |
| 250.14 | Type 1 diabetes with neurological manifestations | 3.61 | 2.3-5.6 |
| 242.1 | Graves' disease | 2.36 | 1.7-3.2 |
| 242 | Thyrotoxicosis with or without goiter | 1.56 | 1.3-1.9 |
| 362.4 | Retinal vascular changes and abnomalities | 1.70 | 1.3-2.1 |
| 202.24 | Large cell lymphoma | 2.00 | 1.5-2.6 |
| 580.12 | Non-proliferative glomerulonephritis | 3.02 | 1.8-5.0 |
| 250.7 | Diabetic retinopathy | 1.48 | 1.2-1-8 |
| 145.2 | Cancer of tongue | 2.60 | 1.7-4.1 |
| 251.1 | Hypoglycemia | 1.57 | 1.3-1.9 |

**Supplementary Table 2.** **Impact of the presence of coeliac disease on Phecodes associated with HLA-DQ2.5 homozygosity.** The occurrence of the highlighted PheCodes was compared in HLA-DQ2.5 homozygous individuals with vs. without the diagnosis of coeliac disease. Odds ratios (ORs) and the corresponding 95% confidence intervals are shown. Odds ratios (ORs) and the corresponding 95% confidence intervals, adjusted for age, sex and body mass index are shown. Abbreviations: NOS, not otherwise specified.

| **PheCode - number** | **Phecode - name** | **OR** | **95%-CI** |
| --- | --- | --- | --- |
| 250.1 | Type 1 diabetes | **3.13** | 2.0-4.8 |
| 250.13 | Type 1 diabetes with ophthalmic manifestations | 1.66 | 0.6-4.6 |
| 250.11 | Type 1 diabetes with ketoacidosis | **2.93** | 1.02-8.4 |
| 70.4 | Chronic hepatitis | **3.09** | 1.2-8.0 |
| 165 | Cancer within the respiratory system | 0.89 | 0.5-1.8 |
| 165.1 | Cancer of bronchus; lung | 0.76 | 0.4-1.6 |
| 202.2 | Non-Hodgkins lymphoma | **2.77** | 1.6-4.8 |
| 244 | Hypothyroidism | **2.37** | 1.8-3.1 |
| 244.4 | Hypothyroidism NOS | **2.35** | 1.8-3.1 |
| 202 | Cancer of other lymphoid, histiocytic tissue | **2.61** | 1.6-4.4 |
| 250.14 | Type 1 diabetes with neurological manifestations | **5.12** | 1.7-15.3 |
| 242.1 | Graves' disease | 1.15 | 0.3-4.8 |
| 242 | Thyrotoxicosis with or without goiter | 1.30 | 0.6-3.0 |
| 362.4 | Retinal vascular changes and abnormalities | 0.82 | 0.3-2.6 |
| 202.24 | Large cell lymphoma | 2.42 | 0.9-6.2 |
| 580.12 | Non-proliferative glomerulonephritis | 0 | 0 |
| 250.7 | Diabetic retinopathy | 2.06 | 1.0-4.3 |
| 145.2 | Cancer of tongue | 0 | 0 |
| 251.1 | Hypoglycemia | 1.96 | 0.9-4.3 |

**Supplementary Table 3. Overview of PheCodes significantly overrepresented in individuals with inflammatory bowel disease.** Only PheCodes enriched in patients with Crohn´s disease and patients with ulcerative colitis but not in coeliac disease are shown.  Odds ratios indicate the occurrence of corresponding PheCodes in subjects with Crohn´s disease (CD) or ulcerative colitis (UC) compared to reference group that carries none of the analysed intestinal disorders. Abbreviations: OR, odds ratio; CD, Crohn´s disease; UC, ulcerative colitis

| Phenotype  number | OR CD | OR UC | Description |
| --- | --- | --- | --- |
| 8.52 | 5.04 | 4.60 | Intestinal infection due to C. difficile |
| 8.6 | 4.27 | 4.71 | Viral Enteritis |
| 41.2 | 4.66 | 2.32 | Streptococcus infection |
| 80 | 4.16 | 2.93 | Postoperative infection |
| 81 | 2.55 | 2.23 | Infection/inflammation of internal prosthetic device; implant; and graft |
| 153 | 2.29 | 2.85 | Colorectal cancer |
| 153.2 | 2.20 | 2.86 | Colon cancer |
| 198 | 1.54 | 1.50 | Secondary malignant neoplasm |
| 275.3 | 7.13 | 2.95 | Disorders of magnesium metabolism |
| 275.5 | 4.56 | 2.05 | Disorders of calcium/phosphorus metabolism |
| 275.53 | 5.97 | 3.07 | Disorders of phosphorus metabolism |
| 280.2 | 4.04 | 4.18 | Iron deficiency anemia secondary to blood loss (chronic) |
| 284 | 6.29 | 3.30 | Aplastic anemia |
| 285.2 | 5.90 | 2.80 | Anemia of chronic disease |
| 287 | 2.60 | 1.90 | Purpura and other hemorrhagic conditions |
| 287.3 | 2.55 | 1.84 | Thrombocytopenia |
| 289.4 | 2.84 | 1.63 | Lymphadenitis |
| 290 | 1.89 | 1.51 | Delirium dementia and amnestic and other cognitive disorders |
| 318 | 2.91 | 1.31 | Tobacco use disorder |
| 351 | 1.50 | 1.39 | Other peripheral nerve disorders |
| 371 | 2.42 | 1.85 | Inflammation of the eye |
| 371.1 | 5.26 | 2.85 | Uveitis, noninfectious or NOS |
| 401 | 2.99 | 2.15 | Hypertension |
| 401.2 | 2.87 | 2.20 | Hypertensive heart and/or renal disease |
| 401.22 | 3.31 | 2.03 | Hypertensive chronic kidney disease |
| 415 | 2.24 | 1.77 | Pulmonary heart disease |
| 427.7 | 2.90 | 2.16 | Tachycardia NOS |
| 441 | 5.93 | 5.32 | Vascular insufficiency of intestine |
| 458.1 | 2.46 | 2.04 | Orthostatic hypotension |
| 479 | 1.94 | 1.75 | Other upper respiratory disease |
| 509 | 3.24 | 1.74 | Respiratory failure, insufficiency, arrest |
| 516 | 2.43 | 1.87 | Abnormal sputum |
| 516,1 | 2.54 | 1.88 | Hemoptysis |
| 568 | 9.97 | 4.69 | Other disorders of peritoneum |
| 568,1 | 10.37 | 4.84 | Peritoneal adhesions (postoperative) (postinfection) |
| 569,2 | 20.23 | 10.32 | Gastrointestinal complications |
| 571,51 | 4.89 | 4.03 | Cirrhosis of liver without mention of alcohol |
| 571,8 | 3.26 | 2.39 | Liver abscess and sequelae of chronic liver disease |
| 571,81 | 3.75 | 3.31 | Portal hypertension |
| 575,1 | 3.38 | 7.73 | Cholangitis |
| 579 | 5.01 | 2.60 | Other symptoms involving abdomen and pelvis |
| 579,2 | 5.13 | 3.17 | Splenomegaly |
| 580,1 | 4.71 | 2.42 | Glomerulonephritis |
| 585,31 | 4.94 | 3.07 | Renal dialysis |
| 586 | 2.49 | 2.41 | Other disorders of the kidney and ureters |
| 586,2 | 2.50 | 2.47 | Cyst of kidney, acquired |
| 590 | 4.76 | 3.24 | Pyelonephritis |
| 594 | 2.91 | 1.98 | Urinary calculus |
| 594,1 | 2.99 | 2.08 | Calculus of kidney |
| 594,2 | 3.63 | 2.63 | Calculus of lower urinary tract |
| 594,3 | 2.78 | 1.92 | Calculus of ureter |
| 595 | 2.27 | 1.90 | Hydronephrosis |
| 614 | 2.23 | 1.75 | Inflammatory diseases of female pelvic organs |
| 619 | 1.63 | 1.59 | Noninflammatory female genital disorders |
| 695,9 | 3.41 | 3.64 | Unspecified erythematous condition |
| 696,42 | 4.41 | 2.57 | Psoriatic arthropathy |
| 701,5 | 4.90 | 2.85 | Abnormal granulation tissue |
| 715 | 3.37 | 2.56 | Other inflammatory spondylopathies |
| 750 | 5.07 | 2.37 | Digestive congenital anomalies |
| 750,2 | 7.88 | 3.56 | Lower gastrointestinal congenital anomalies |
| 750,21 | 7.11 | 4.48 | Congenital anomalies of intestine |
| 783 | 2.74 | 1.73 | Fever of unknown origin |
| 853 | 30.99 | 28.20 | Complication of colostomy or enterostomy |
| 854 | 3.77 | 2.19 | Complications of cardiac/vascular device, implant, and graft |
| 962,3 | 5.85 | 4.06 | Hormones and synthetic substitutes causing adverse effects in therapeutic use |
| 969 | 2.41 | 2.10 | Poisoning by psychotropic agents |
| 994 | 3.19 | 2.29 | Sepsis and SIRS |
| 994,2 | 3.19 | 2.29 | Sepsis |

**Supplementary Table 4. ICD-10 diagnosis for primary cause of death for patients wihe Coeliac disease (CeD), Ulcerative Colitis (UC) and Crohn´s disease (CD)**

| **ICD-10** | **Description** | **CeD** | **UC** | **CD** |
| --- | --- | --- | --- | --- |
|  |  |  |  |  |
| C00-C14 | Malignant neoplasms of lip, oral cavity and pharynx | 2 | 1 | 2 |
| C15-C26 | Malignant neoplasms of digestive organs | 39 | 69 | 43 |
| C30-C39 | Malignant neoplasms of respiratory and intrathoracic organs | 11 | 30 | 22 |
| C40-C41 | Malignant neoplasms of bone and articular cartilage | 0 | 1 | 0 |
| C43-C44 | Melanoma and other malignant neoplasms of skin | 6 | 6 | 0 |
| C45-C49 | Malignant neoplasms of mesothelial and soft tissue | 3 | 11 | 2 |
| C50 | Malignant neoplasm of breast | 4 | 14 | 5 |
| C51-C58 | Malignant neoplasms of female genital organs | 12 | 10 | 4 |
| C60-C63 | Malignant neoplasms of male genital organs | 8 | 13 | 4 |
| C64-C68 | Malignant neoplasms of urinary tract | 9 | 13 | 3 |
| C69-C72 | Malignant neoplasms of eye, brain and other parts of central nervous system | 4 | 6 | 4 |
| C73-C75 | Malignant neoplasms of thyroid and other endocrine glands | 1 | 0 | 1 |
| C76-C80 | Malignant neoplasms of ill-defined, secondary and unspecified sites | 11 | 16 | 10 |
| C81-C96 | Malignant neoplasms, stated or presumed to be primary, of lymphoid, haematopoietic and related tissue | 18 | 15 | 8 |
| C97 | Malignant neoplasms of independent (primary) multiple sites | 1 | 0 | 0 |
|  |  |  |  |  |
| K20-K31 | Diseases of oesophagus, stomach and duodenum | 2 | 3 | 1 |
| K35-K38 | Diseases of appendix | 0 | 0 | 0 |
| K40-K46 | Hernia | 0 | 1 | 1 |
| K50-K52 | Noninfective enteritis and colitis | 0 | 9 | 16 |
| K55-K64 | Other diseases of intestines | 6 | 7 | 3 |
| K65-K67 | Diseases of peritoneum | 0 | 0 | 0 |
| K70-K77 | Diseases of liver | 4 | 6 | 5 |
| K80-K87 | Disorders of gallbladder, biliary tract and pancreas | 1 | 11 | 3 |
| K90-K93 | Other diseases of the digestive system | 4 | 0 | 1 |
|  |  |  |  |  |
| I05-I09 | Chronic rheumatic heart diseases | 1 | 4 | 1 |
| I10-I15 | Hypertensive diseases | 1 | 4 | 1 |
| I20-I25 | Ischaemic heart diseases | 29 | 46 | 25 |
| I26-I28 | Pulmonary heart disease and diseases of pulmonary circulation | 0 | 2 | 0 |
| I30-I52 | Other forms of heart disease | 10 | 5 | 7 |
| I60-I69 | Cerebrovascular diseases | 8 | 17 | 14 |
| I70-I79 | Diseases of arteries, arterioles and capillaries | 0 | 6 | 2 |
| I80-I89 | Diseases of veins, lymphatic vessels and lymph nodes, not elsewhere classified | 2 | 4 | 0 |
|  |  |  |  |  |
| J09-J18 | Influenza and pneumonia | 7 | 5 | 7 |
| J20-J22 | Other acute lower respiratory infections | 1 | 1 | 1 |
| J30-J39 | Other diseases of upper respiratory | 0 | 0 | 0 |
| J40-J47 | Chronic lower respiratory diseases | 11 | 14 | 10 |
| J60-J70 | Lung diseases due to external agents | 0 | 2 | 1 |


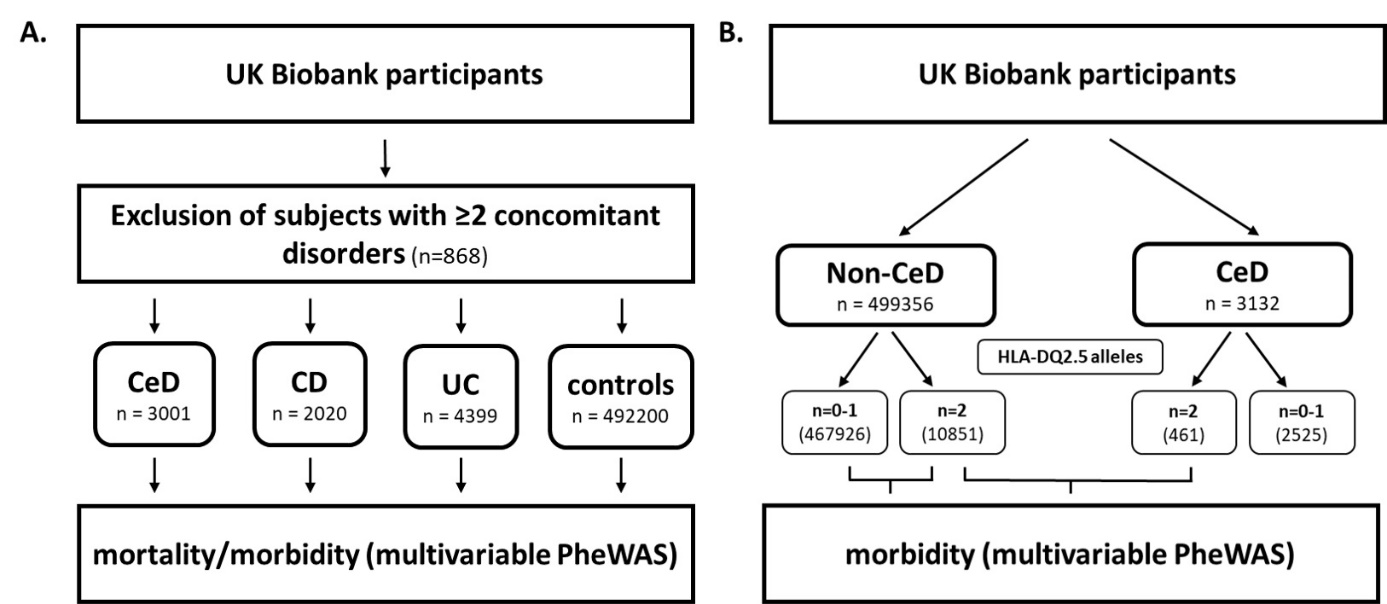


**Supplementary Figure 1. Overview of the analysed cohorts.** Flow charts illustrate the cohorts that were used for analysis of mortality and morbidity associated with chronic intestinal disorders (A) as well as the importance of the major genetic predisposition factor, i.e. HLA-DQ2.5 homozygosity, for coeliac disease (CeD)-related morbidity (B).Abbreviations: CD, Crohn's disease; UC, ulcerative colitis.


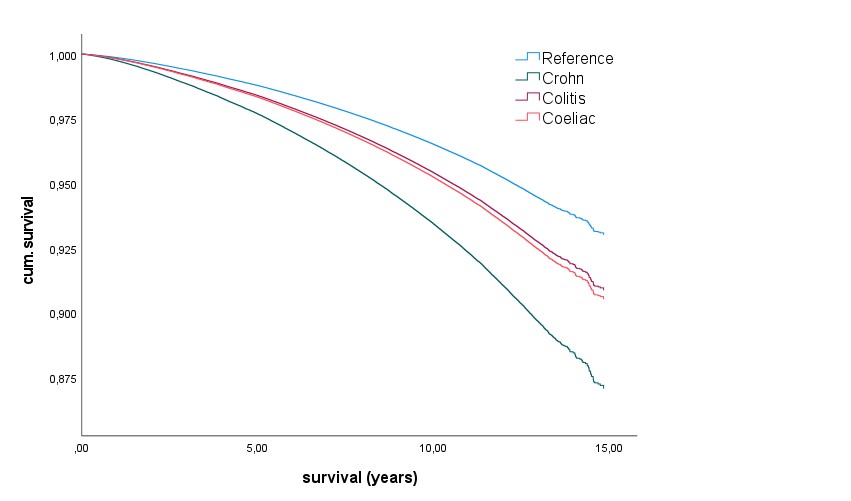


**Supplementary Figure 2.** **Cumulative survival in individuals with analysed intestinal disorders.** Individuals with Crohn´s disease (Crohn), ulcerative colitis (Colitis) and coeliac disease (Coeliac) were compared to the reference group that carries none of these conditions.


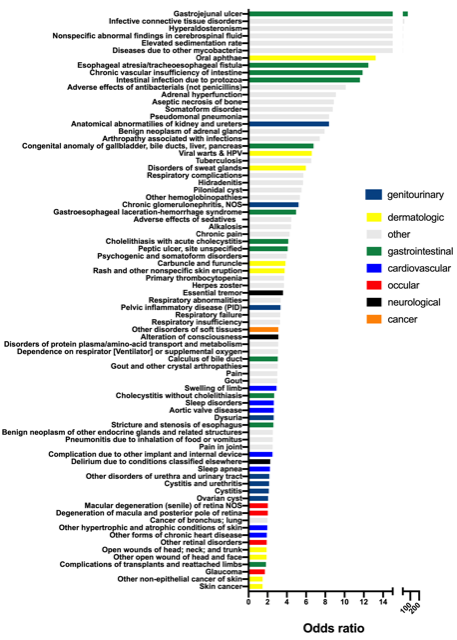


**Supp. Figure 3.**  **Overview of PheCodes significantly overrepresented in individuals with Crohn´s disease.** Only PheCodes enriched in patients with Crohn´s disease but not in coeliac disease or ulcerative colitis are shown. Odds ratios indicate the occurrence of corresponding PheCodes in subjects with Crohn´s disease compared to reference group that carries none of the analysed intestinal disorders. Colours highlight the indicated disease classifications of PheCodes.


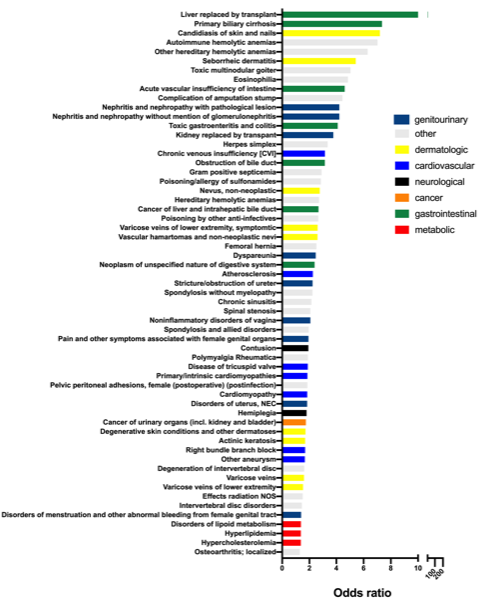


**Supp. Figure 4.** **Overview of PheCodes significantly overrepresented in individuals with ulcerative colitis.** Only PheCodes enriched in patients with ulcerative colitis but not in Crohn's disease or coeliac disease are shown. Odds ratios indicate the occurrence of corresponding PheCodes in subjects with ulcerative colitis compared to reference group that carries none of the analysed intestinal disorders. Colours highlight the indicated disease classifications of PheCodes.
